# Functional neuroanatomy of the rhinophore of *Aplysia punctata*

**DOI:** 10.1186/1742-9994-3-6

**Published:** 2006-04-06

**Authors:** Adrian Wertz, Wolfgang Rössler, Malu Obermayer, Ulf Bickmeyer

**Affiliations:** 1 Biologische Anstalt Helgoland, Alfred Wegener Institute for Polar and Marine Research in Helmholtz Society, Kurpromenade 201, 27483 Helgoland, Germany; 2 Behavioural Physiology and Sociobiology, Biocenter, University of Würzburg, Am Hubland, 97074 Würzburg, Germany; 3 Max Planck Institute of Neurobiology, Department of Systems and Computational Neurobiology, Am Klopferspitz 18, 82152 Martinsried, Germany

## Abstract

**Background:**

For marine snails, olfaction represents a crucial sensory modality for long-distance reception, as auditory and visual information is limited. The posterior tentacle of *Aplysia*, the rhinophore, is a chemosensory organ and several behavioural studies showed that the rhinophores can detect pheromones, initiate orientation and locomotion toward food. However the functional neuroanatomy of the rhinophore is not yet clear. Here we apply serotonin-immunohistochemistry and fluorescent markers in combination with confocal microscopy as well as optical recording techniques to elucidate the structure and function of the rhinophore of the sea slug *Aplysia punctata*.

**Results:**

With anatomical techniques an overview of the neuroanatomical organization of the rhinophore is presented. Labelling with propidium iodide revealed one layer of cell nuclei in the sensory epithelium and densely packed cell nuclei beneath the groove of the rhinophore, which extends to about two third of the total length of the rhinophore. Serotonin immunoreactivity was found within the olfactory glomeruli underneath the epithelium as well as in the rhinophore ganglion. Retrograde tracing from the rhinophore ganglion with 4-(4-(dihexadecylamino)styryl)-*N*-methylpyridinium iodide (DiA) demonstrated the connection of glomeruli with the ganglion. Around 36 glomeruli (mean diameter 49 μm) were counted in a single rhinophore. Fluorimetric measurements of intracellular Ca^2+ ^levels using Fura-2 AM loading revealed Ca^2+^-responses within the rhinophore ganglion to stimulation with amino acids. Bath application of different amino acids revealed differential responses at different positions within the rhinophore ganglion.

**Conclusion:**

Our neuroanatomical study revealed the number and position of glomeruli in the rhinophore and the rhinophore ganglion as processing stage of sensory information. Serotonin-immunoreactive processes were found extensively within the rhinophore, but was not detected within any peripheral cell body. Amino acids were used as olfactory stimuli in optical recordings and induced sensory responses in the rhinophore ganglion. The complexity of changes in intracellular Ca^2+^-levels indicates, that processing of odour information takes place within the rhinophore ganglion. Our neuroanatomical and functional studies of the rhinophore open up a new avenue to analyze the olfactory system in *Aplysia*.

## Background

Chemical signal perception represent an important part of communication and is used by a large variety of organisms from protozoans [1] and yeast [2] to insects [3], molluscs [4,5], fish [6], mammals [7] and humans [8]. Sea slugs of the genus *Aplysia *have been investigated intensively with respect to behavioural and neurobiological studies, and the gill withdrawal reflex has become a well known neuronal model circuit for studies of the cellular basis of learning and memory [9-11]. Fewer studies have targeted the olfactory system of *Aplysia *[12-14]. In *Aplysia*, chemosensation plays an important role in the context of various behaviours, e.g. localization of food [14,15] and sexual behaviour [16]. The rhinophore is considered as the olfactory organ in *Aplysia *[15] and was suggested to be important for the detection of pheromones [16,17]; different peptide-pheromones were identified in the genus *Aplysia *[4,18,19]. Recently, it could be demonstrated that secondary metabolites (alkaloids) from marine sponges stimulate neurons in the rhinophore ganglion of the rhinophore of *Aplysia punctata *[20].

Despite biological significance of olfaction, little is known about structural and functional aspects of the olfactory sensory pathway in *Aplysia*. The neuroanatomy of the tentacle was investigated in the terrestrial snail *Achatina *[21], which belong to the group of Pulmonata and to stylommatophoran snails. The tentacles of *Achatina fulica *contain a tentacle ganglion, glomeruli and four pathways for the projection of olfactory sensory neurons were described. *Aplysia *was shown to posses a rhinophore ganglion but in contrast to *Achatina *the eye is at the base and not at the top of the rhinophore, and photoreceptors are located in the rhinophore epithelium [21-24]. In *Phestilla sibogae *the presence of serotonin, dopamine and norepinephrine in the rhinophores was confirmed [25], possibly revealing glomerulus-like structures along the olfactory pathway [26]. Serotonin-immunoreactive elements were also found in the rhinophores of *Pleurobranchea californica *and *Tritonia diomedea *[27]. *Phestilla sibogae*, *Pleurobranchea californica *and *Tritonia diomedea *are closely related to *Aplysia punctata *belonging to the systematic group of ophistobranchia. Here we look for serotonin immunoreactivity in the rhinophore of *Aplysia punctata*. In addition, neuroanatomical tracing and histology are used to analyse the structure of the rhinophore.

Amino acids were shown to be potent olfactory stimuli for aquatic animals [28-31] and elicit feeding responses in *Pleurobranchaea californica *[32]. Murphy and Hadfield [33] studied the innervation of the rhinophores and the oral tentacles of *Phestilla sibogae *and used electrophysiological techniques to demonstrate that only the rhinophores were highly selective to free amino acids. Therefore we use optical imaging technique and amino acids as stimuli, to investigate chemoreceptive processing in the rhinophore. The present study is aimed to contribute to our understanding of the olfactory sensory pathway in marine snails and the chemosensory capabilities of these animals.

## Results and discussion

### Neuroanatomy of the rhinophore

**Figure 1:**
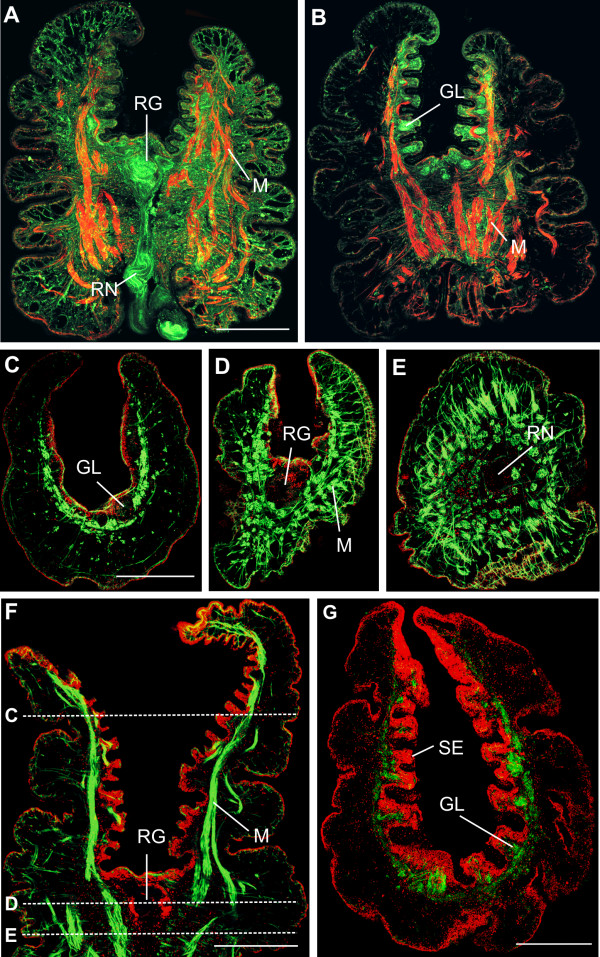
**Anatomical overview of the rhinophore**. Neuroanatomy of the rhinophore: confocal images of sections labelled with an antibody to serotonin, fluophore-conjugated phalloidin and propidium iodide. A, B: Sagittal sections of the rhinophore at different planes. F-actin labelling with phalloidin (red) revealed the course of muscle fibres (M). Serotonin-immunoreactivity (green) is present in the rhinophore nerve (RN), the rhinophore ganglion (RG) and the glomeruli (GL). C, D, E, F: Sections labelled with phalloidin (green) and propidium iodide (red). C, D, E are cross sections at different planes at the positions indicated in F. In the upper part of the rhinophore glomeruli are situated beneath the epithelium. The rhinophore ganglion is situated at the basis of the groove. G: Nuclear labelling with propidium iodide (red) reveals the inner sensory epithelium (SE). Serotonin immunoreactivity (green) is present in the Glomeruli (GL). Scale bars = 500 μM (scale bar in A is also valid for B, in C is also valid for D and E).

The anatomy of the rhinophore of *Aplysia *was previously investigated only with respect to the location of sensory cells [13] and as part of a phylogenetic study of seahares [34]. The presence of neuromodulators such as catecholamines has been demonstrated in the rhinophore of *Aplysia californica *by Croll [35]. This is the first study to focus on the functional neuroanatomy of the rhinophore of *Aplysia punctata *with respect to number and location of glomeruli, serotonergic innervation and neuronal pathways. The rhinophore of *Aplysia punctata *contains a groove, which extends to about two third of the total length of the rhinophore. Figures 1A and 1B show an example of a longitudinally sectioned rhinophore labelled with phalloidin and serotonin-immunoreactivity (IR). The rhinophore usually contracted during the process of dissection, and prominent longitudinal muscles became strongly labelled with phalloidin binding to muscular f-actin (Figs 1A, B, F). Serotonin-IR was detectable in various regions of the rhinophore: the rhinophore nerve, the rhinophore ganglion at the basis of the groove, and the glomeruli (Figs 1A, B, G). Serotonergic fibres proceeded from the rhinophore nerve via the rhinophore ganglion to the glomeruli. Since no serotonin-IR was detectable in cell bodies within the rhinophore, all serotonergic neurons innervating the rhinophore should be of extrinsic origin. Croll [35] described tyrosine hydroxylase immunoreactivity in both large and small somata beneath the epithelium in the rhinophore, homogeneously distributed over walls of the entire structure, whereas serotonergic immnureactivity was found in cellular processes but not the somata inside the rhinophore. Croll et al. [26] found no serotonergic cell bodies in the periphery in the nudibranch *Phestilla*, similar to our findings. This indicates a physiological role of catecholamines and serotonin in olfactory processing mediated by centrifugal neurons in the case of serotonin and more local modulation in the case of catecholamines. Serotonergic innervation of olfactory glomeruli is commonly found in insects and was shown to enhance the response of olfactory projection neurons (e.g.: [36,37]).

**Figure 2:**
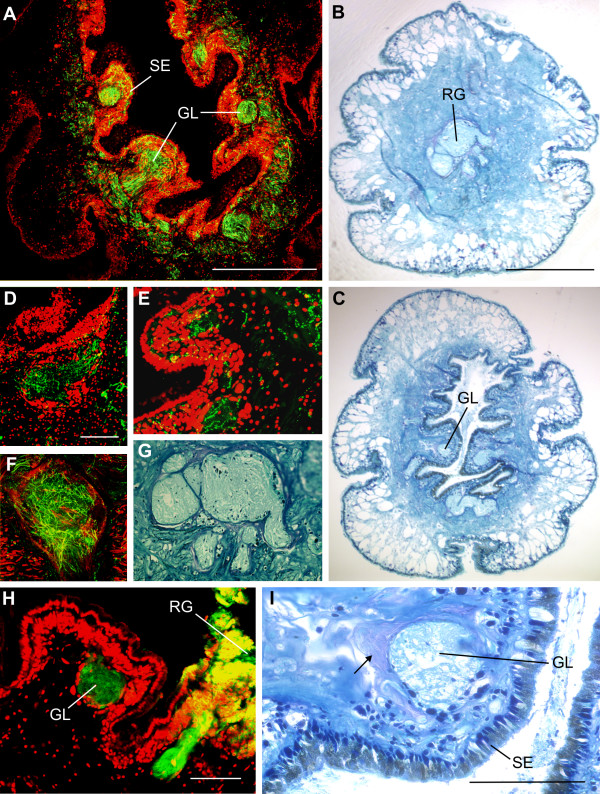
**Anatomical details of the rhinophore**. Neuroanatomy and histology of the rhinophore. A: Cross sections labelled with serotonin-immunoreactivity (green) and propidium iodide (red). Nuclear labelling shows the layers of cell nuclei in the sensory epithelium (SE) around the groove. Glomeruli (GL) are situated beneath the sensory epithelium. Scale bar = 500 μm. B, C, G: Histological cross sections of the rhinophore at different planes along the longitudinal axis (stained with Mallory's stain). The rhinophore ganglion (RG) is folded and located in the middle of the rhinophore. Scale bar = 500 μm. G: Higher magnification of the rhinophore ganglion shown in B. Scale bar = 100 μm. D, E, F,: Serotonin-immunoreactivity is labelled green and cell nuclei are labelled red in D and E, whereas in F phalloidin is labelled in red. The immunoreactivity is showing serotonergic innervation of the rhinophore ganglion (D, F) and the sensory epithelium (E). Cell nuclei in the sensory epithelium labelled with propidium iodide (D, E). F: Light labelling with phalloidin in the rhinophore ganglion. Scale bars for D, E, F, G = 100 μm. H: Injection of DiA (green) in the rhinophore ganglion revealed the connection from the rhinophore ganglion to a glomerulus. The big amount of DiA crystals are fluorescing yellow and indicate thereby the injection location. Labelling with Propidium iodide (red) shows the layers of cell nuclei in the epithelium. I: Histological section (Mallory stain). Individual glomerulus situated beneath the sensory epithelium and surrounded by a glial-like cell layer (arrow).

A series of cross sections demonstrates phalloidin-labelled muscle-fibres bundles oriented in the longitudinal and horizontal axis of the rhinophore (Figs 1C, D, E). Glomeruli were situated beneath the sensory epithelium close to the inner wall of the entire groove (Figs 1B, 2A, C). Staining of cell nuclei with propidium iodide indicates that the glomeruli are not surrounded by a regular border of periglomerular cell somata (Figs 1G, 2A, H). In histological sections, however, glomeruli appeared to be surrounded by a layer of glia-like processes (Fig. 2I). In contrast to insects and vertebrates, where olfactory glomeruli became brightly labelled with phalloidin due to aggregation of neuronal f-actin [38], glomeruli within the rhinophore of *Aplysia punctata *were not labelled with phalloidin, and the rhinophore ganglion was only lightly stained with phalloidin (Fig. 2F). Histological sections revealed that the rhinophore ganglion has a folded structure (Fig. 2G). Retrograde labelling of the glomeruli by insertion of 4-(4-(dihexadecylamino)styryl)-*N*-methylpyridinium iodide (DiA) in the rhinophore ganglion demonstrated the connection between the rhinophore ganglion and the glomeruli (Fig. 2H). DiA stainings show the neuronal connection between the rhinophore ganglion and glomeruli. Retrograde labelling with DiA revealed mostly neuropil staining indicating that neuronal processes extending from the rhinophore ganglion to the glomeruli were preferentially labelled. We do not know if the cell bodies of these neurons lie in the rhinophore ganglion (because of massive staining) or further out in the periphery, or on adjacent sections. Future studies will focus in more detail on individual projections of sensory neurons using patch clamp/single neuron recordings and dye filling techniques.

The number of glomeruli was estimated in a complete series of sections of the rhinophore labelled with 5-HT antibody. The total number of glomeruli counted in one rhinophore was 36 in one rhinophore, and the mean diameter of individual glomeruli averaged 49 μm +/- 27 μm with a range from 25 μm to 135 μm.

Previous investigations on *Aplysia californica *have shown that the groove houses various types of sensory cells [13]. Emery and Audesirk [13] suggested that intraepithelial cells with 30 μM long cilia produce a steady water flow around the epithelial cells of the rhinophore to facilitate olfaction. Anterograde labelling experiments in a terrestrial snail (*Achatina fulica*) by Chase and Tollozcko [21] have shown that sensory neurons project to the glomeruli and directly to the rhinophore ganglion or to further centres in the cerebral ganglion. Future experiments are needed to find out if similar projection patterns of sensory neurons are present in *Aplysia*.

Labelling of cell nuclei with propidium iodide and histological sections showed the high amount of cell nuclei in the sensory epithelium (Figs 1F, G, 2A, H, I). The layer of sensory cell nuclei is clearly separated from the more basal nuclei (Fig 2H). Glomeruli were located underneath this epithelium (Figs 2H, I).

Propidium Iodide and Mallory's stain revealed cell nuclei of variable sizes surrounding the glomeruli. Both methods revealed very few nuclei inside the glomeruli similar to the conditions in vertebrates and in insects (Figs. 2H, I). Propidium Iodide has very high affinity to nucleic acids, and since we used agarose sections with excellent penetration properties we are convinced that all nuclei were labelled. In contrast to the glomeruli, cell nuclei were present inside the rhinophore ganglion (Fig. 2G). Cell nuclei can be from both neuronal and glial cell bodies.

The anatomy of the rhinophores with distributed glomeruli around the groove leaves different possibilities open for central projections of olfactory receptor neurons (ORNs). The general view of a uniglomerular projection pattern, which seems to occur in most insects [39,40] and vertebrates [41,42], would be complicated with the present arrangement of glomeruli. Possibly, ORNs of the same type are situated beneath one glomerulus and are not uniformly distributed over the sensory epithelium, or, in the other case, axonal projections of individual ORNs to their target glomeruli would be very long and across the rhinophore. Recent studies in *Xenopus laevis *described an innervation of more than one glomerulus by individual ORNs [43]. Multiglomerular projection patterns also appear in crustaceans [44]. In *Aplysia*, future studies on the projection pattern of ORNs combined with functional imaging studies of the glomeruli will provide further insight in the functional role of glomeruli in molluscs.

### Calcium imaging of odour evoked responses within the rhinophore ganglion

**Figure 3:**
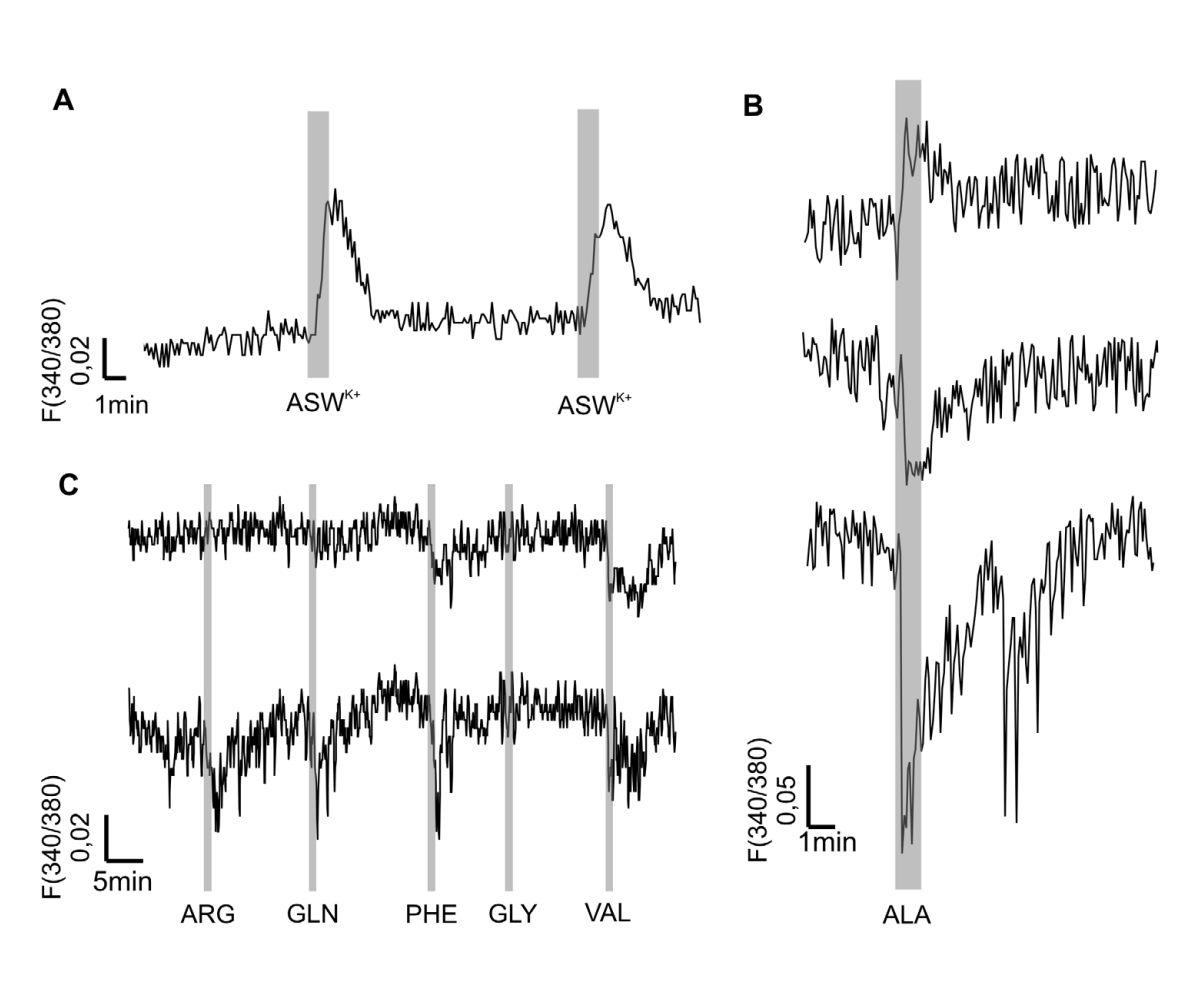
**Fluorimetric measurements**. Measurement of changes of Ca^2+^-levels in separated ROIs in response to high K^+ ^and amino acids in the rhinophore ganglion (stimulus duration was one minute in each case). **A**: Application of artificial sea water with high K^+^. Responses were stable and could be reproduced after wash-out. **B**: Ca^2+^-responses of ROIS of three different animals to the stimulation with 2 mM alanine (ALA). An elevation as well as a decrease of the Ca^2+^-levels were found. **C**: Responses in two regions to stimulation with different amino acids (2 mM). Arginine (ARG) and glutamine (GLN) did not induce a response in one region (upper trace), whereas glycine (GLY) and valine (VAL) did evoke a response. Phenylalanine (PHE) did not induce any response. A second region (lower trace) responded to all amino acids except PHE.

To investigate whether the rhinophore ganglion responds to olfactory stimulation of the sensory epithelium of the rhinophore, we applied different amino acids as odorants and recorded the responses optically. First of all we tested our system by the application of artificial sea water (ASW) with high K^+ ^(Fig. 3A). The Ca^2+^-response induced by a high K^+^-solution could be reproduced and showed nearly a similar increase after repetitive application.

We chose different amino acids, as they were used in previous experiments in gastropod molluscs [32,33]. To avoid direct excitation of neurons within the rhinophore by the stimuli, we did not use glutamic and aspartic acid, which induced highest responses in the nudibranch *Phestilla sibogae *[33]. In more than 10 experiments we found no Ca^2+ ^– response within the rhinophore ganglion induced by the application of methionin at different concentration (200 μm – 20 mM).

Clear Ca^2+^-responses within the rhinophore ganglion were found during stimulation with alanine (ALA). Figure 3B shows three responses to ALA from three different animals. In all experiments 2 mM ALA was applied. We found a decrease of Ca^2+^-levels as well as an increase (Fig 3B upper trace). Application of different amino acids (2 mM each) during one experiment revealed differential responses of intracellular Ca^2+^-levels in distinct regions (Fig 3C). The traces in Fig 3C show Ca^2+^-measurements in two different regions of interest. Arginine (ARG) and glutamine (GLN) induced a response in one region, whereas the other region showed no response, but both regions responded with a change in Ca^2+^-level to the stimulation with glycine (GLY) and valine (VAL). Both regions showed a decrease in Ca^2+^-levels to all stimuli (Fig. 3C) indicating an inhibition or reduction of cellular activity by these stimuli. Stimulation with 2 mM phenylalanine (PHE) induced no detectable responses.

**Figure 4:**
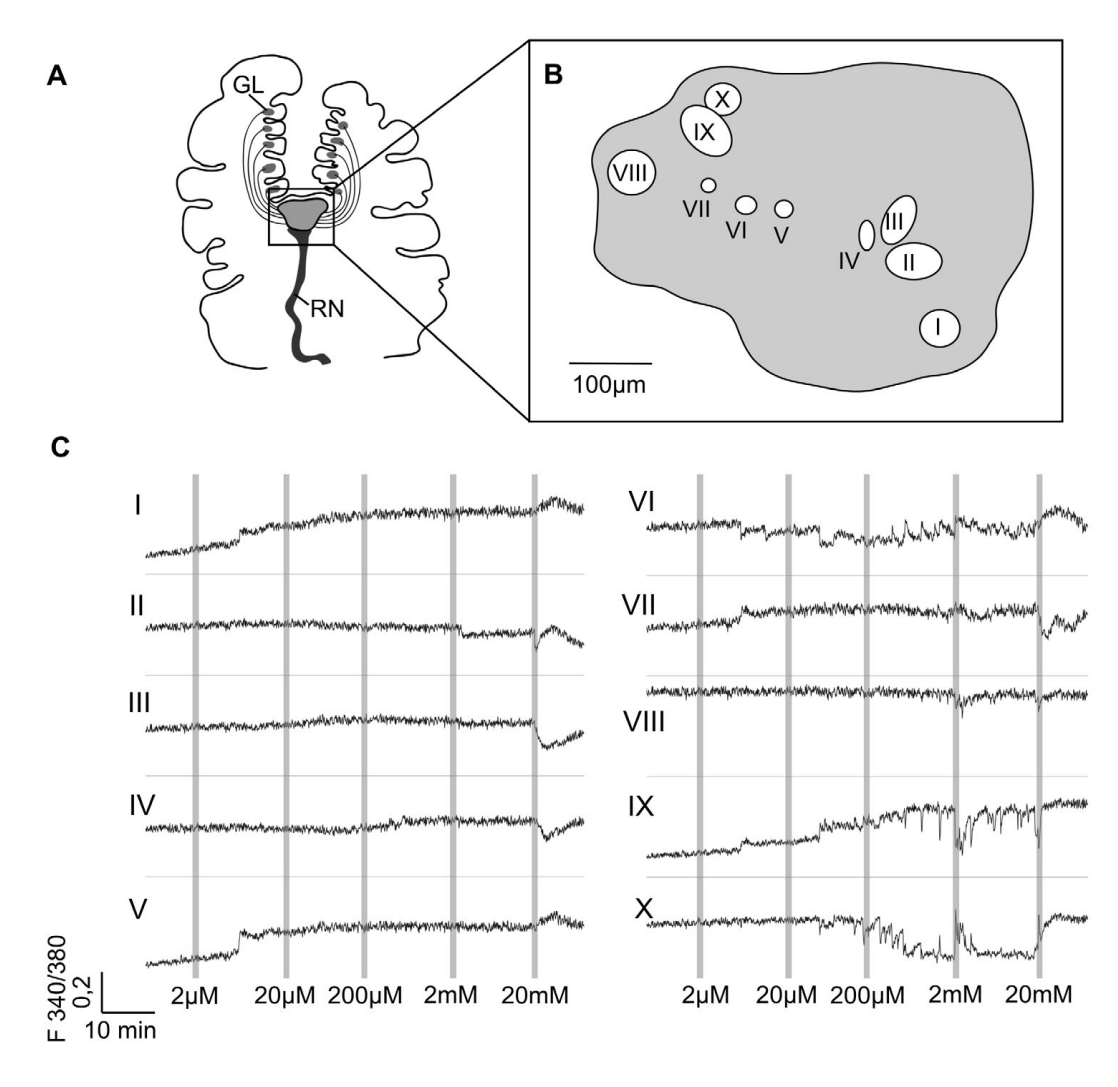
**Ca^2+^-Responses of ROIs to different concentrations of alanine**. **A**: Schematic overview of the rhinophore of *Aplysia punctata*: Measurements were performed within the rhinophore ganglion (box). Glomeruli (GL) with putative projections to the ganglion. Rhinophore nerve (RN). **B**: Locations of ten regions within the rhinophore ganglion showing differential responses to stimulation with the amino acid alanine (ALA). Scale bar = 100 μm. **C**: Ca^2+^-responses to alanine (2 μM – 20 mM) recorded from the regions indicated in (stimulus duration was one minute in each case) B. The application of 20 mM ALA induced in all ROIs a response, a decrease or an increase of the Ca^2+^-levels. To 2 mM ALA clear responses were found in regions VIII, IX and X. For the lower ALA concentrations no clear stimulus dependent response could be ascertained.

The responses to different concentrations of ALA (2 μM – 20 mM) were highly dependent on the recording position within the ganglion. Ten responses from ten regions within the rhinophore ganglion are demonstrated in figure 4. Regions of interest (ROIs) were selected more or less randomly across the rhinophore ganglion at the beginning of an experiment, and only responsive regions are shown in the figures. The size of individual ROIs can be estimated from the scale bar (Fig. 4B). We assume that in many cases ROIs may have not included a single neuron since the diameter ROIs were between 15 and 60 μM and only the largest cell bodies reach 50 μM.

In the course of a single experiment, odour induced responses to amino acids recorded from different regions remained similar. We applied ALA in concentration from 2 μm to 20 mM. At low concentration (<2 mM) it was difficult to decide whether an observed Ca^2+^-response was stimulus dependent. At a concentration of 2 mM ALA, clear responses could be observed in three regions (Fig 4C VIII-X). Regions VIII and IX showed a decrease of the Ca^2+^-levels, whereas in region X the Ca^2+^-level increased.

In response to 20 mM ALA all of the selected regions showed a change in intracellular Ca^2+^-levels. It was either a decrease or an increase of the Ca^2+^-levels, or even a combination of both as shown in trace II. Remarkably, responses from neighbouring regions (II and III; IX and X) showed opposing Ca^2+^-responses indicating a possible inhibitory coupling. The spatial response patterns were complex. Stimulation with 20 mM ALA induced an increase in Ca^2+^-levels in four regions (Fig. 3A-I,-V,-VI,-X), whereas in five other regions we found a decrease in Ca^2+^-levels (Fig. 3A-III,-IV,-VII,-VIII,-IX), and in region II a decrease was followed by an increase in Ca^2+^-levels.

In all of our experiments, the highest Ca^2+^-responses were induced by ALA. Since the rhinophore is cut during the preparation, we cannot exclude partial injury of the rhinophore ganglia. However, the calcium imaging experiments demonstrate that parts of the rhinophore ganglion respond differentially to stimulation of the sensory epithelium with amino acids indicating that olfactory stimuli are relayed and processed in the rhinophore ganglion. In a previous investigation we tested sponge alkaloids and revealed clear responses to sceptrin at 200 μM concentration [20]. The term olfaction is used in terrestrial animals for airborne chemicals, but also in aquatic animals for water borne chemicals detected by primary receptor neurons in the nose (e.g. fish, amphibians) or on the antennae (crustaceans) [45]. In vertebrates it is referred to as long distance reception in contrast to close range chemoreception via taste receptors (secondary receptor cells). In *Phestilla sibogae *different densities of subepithelial sensory cells and intraepithelial sensory cells were found in the oral tentacle and the rhinophores [46]. These morphological data together with the electrophysiological evidence for greater sensitivity of the rhinophores [33] led Boudko and colleagues [46] to the conclusion, that rhinophores serve for long-distance chemoreception or olfaction. To our knowledge, a clear separation based on receptor cell morphology has not been done for *Aplysia*; therefore we prefer to use the term "olfaction" because of the apparent similarities regarding the organization of the central pathway in glomeruli.

In *Limax*, processing of olfactory information was mainly investigated at the level of the procerebrum [47-49], and complex odour induced oscillations were described. In *Aplysia*, using calcium imaging techniques we did not find any indications for odour induced oscillations at the level of the rhinophore ganglion. However, the complexity of changes in intracellular Ca^2+^-levels indicates that processing of odour information takes place within the rhinophore ganglion.

The spatial distribution of odour induced activity recorded in this study indicates that the activity in the rhinophore ganglion depends on the chemical nature and intensity of the stimulus.

Various studies in insects as well as vertebrates suggest that olfactory glomeruli represent functional units for odour processing, and olfactory information is represented in a spatial map of differentially activated glomeruli (e.g.:[50,51]). It will be interesting to determine if this is also the case in *Aplysia *and if glomeruli represent functional units for odour processing. Glomeruli diameters ranged between 25 μm and 135 μm indicating different numbers of neurons converging in one glomerulus. In insects, macroglomeruli were found to play an important role in pheromone communication (e.g.: [52,53]). As the rhinophore of *Aplysia *plays an important role in pheromone detection [17], it would be interesting to investigate the large glomeruli and their potential function in pheromone processing. Measuring the responses of sensory neurons in the epithelium may also help to identify olfactory sensory neurons. Many marine organisms produce secondary metabolites for defence, deterrence and as pheromones. Sea slugs as well as many other marine animals depend on chemosensory information from their aquatic environment, as the optical sense plays a minor role in shallow muddy waters or the deep sea and acoustic/mechano-sensory senses give only limited information about for example food quality. The chemical sense probably is the primary sense in many marine organisms, and *Aplysia *represents a promising model system for future investigations of the chemical ecology of sea slugs.

## Conclusion

The glomeruli and the rhinophore ganglion represent different processing stages for sensory information. We found 36 glomeruli in the rhinophore and retrograde labelling with DiA revealed the connection between glomeruli and rhinophore ganglion. The glomeruli are situated close to the epithelials layers towards the lumen of the groove and appear to be surrounded by a thin glia-like sheath. Serotonin-immunoreactivity was found extensively within the rhinophore, but was not detected in any peripheral cell body. Therefore these serotonergic fibres appear to be efferent projections from central ganglia. Amino acids were used as potentially important olfactory stimuli for aquatic animals, and optical recordings show that amino acids induce sensory responses in the rhinophore. The complexity of changes in intracellular Ca^2+^-levels led us suggest, that processing of odour information takes place within the rhinophore ganglion. Our neuroanatomical and functional studies of the rhinophore open up a new avenue to analyze the olfactory system in *Aplysia*, leading towards understanding neuronal processing of chemical cues in the marine environment.

## Methods

### Tissue preparation, immunocytochemistry, and fluorescent tracers

Specimens of *Aplysia punctata *were collected from shallow waters around Helgoland. Animals were cooled in ice, fixed in 4% formaldehyde in Artificial Sea Water (ASW; pH 7.5; in mM: 460 NaCl, 104 KCl, 55 MgCl, 11 CaCl_2_, and 15 Na -HEPES (N-(2-hydroxyethyl) piperazine-N-2-ethansulfonic acid Na-salt). Before further treatments rhinophores were washed three times in 0.1 M phosphate buffered saline (PBS; pH 7.2). For labelling with fluophore-conjugated phalloidin, immunocytochemistry, tracing with lipophilic markers and nuclear staining, the rhinophores were embedded in 5% low-melting point agarose (Agarose II, Amresco, Solon, OH, No. 210–815) and sectioned in a frontal or sagittal plane at 150 μm thickness with a vibrating microtome (Leica VT 1000S, Wetzlar, Germany). Free-floating agarose sections were preincubated in PBS with 0.2% Triton X-100 and 2% normal goat serum (ICN, Biomedicals, Orsay, France, Cat. No.191356) for one hour at room temperature. Different combinations of double stainings were performed. To label serotonergic neurons, sections were incubated with a primary antibody against 5-HT, raised in rabbit (1:4000, DiaSorin, Stillwater, MN, Cat. No. 20080, Lot No. 051007) in PBS with 0.2% Triton X-100 and 2% NGS overnight at room temperature. This antibody was used in previous studies in gastropod molluscs [26,27]. After five rinses in PBS, sections were incubated in Alexa Fluor 488 conjugated goat anti-rabbit secondary antibody (1:250, Molecular Probes, Eugene, OR, Cat. No. A -11008). To label filamentous (f)-actin in muscles and neurons, sections were incubated in 0.2 units of Alexa Fluor 488 or 568 phalloidin (Molecular probes, A-12379 and A-12380) in PBS overnight at 4°C. To stain cell nuclei, sections were incubated for 15 min in 25 μg/ml propidium iodide (Molecular probes, P-1304) in PBS with 0.2% Triton X-100 at room temperature. Sections were finally washed at least five times with PBS, transferred into 60% glycerol/PBS for 30 min, and mounted on slides in 80% glycerol in PBS.

### Staining with lipophilic tracers

The fluorescent lipophilic dye 4-(4-(dihexadecylamino)styryl)-*N*-methylpyridinium iodide (DiA; Molecular Probes, D -3883) was used as retrograde tracer. Crystals of DiA were transferred into the rhinophore ganglion with a fine minuten pin. Following dye application the rhinophores were fixed in 4% formaldehyde in PBS and incubated for 7 days at 4°C. Embedding, sectioning and double labelling with propidium iodide were performed as described above. Finally, sections were rinsed in PBS and mounted in PBS on microscopic slides.

### Histology, Mallory's stain and microscopy

Rhinophores were fixed in Bouin's fixative solution for 2 days, washed with ethanol, embedded in Spurr's resin and sectioned in a frontal plane (6 μm). After normal histological procedures, the plastic sections were stained after the method from Mallory (for details [54]) on a hotplate. The sections were washed with distilled water, dried on the hotplate and mounted in Entellan (Merck,. Darmstadt, Germany). Images were taken with a digital Camera (Spotinsight Color, Vistron Systems, Puchheim, Germany) mounted on a microscope (Zeiss Axiophot, Carl Zeiss GmbH, Jena, Germany). Image processing was performed with CorelDRAW Graphics Suite (Corel Corporation, Ottawa, Ontario, Canada).

Fluorescent tracers and antibodies were visualized using a laser-scanning confocal microscope (Leica TCS SP). Glomeruli counts per slice were corrected for double-counting in adjacent slices using the method after Weibel [55]. Image processing was performed with the following software: Zeiss Image Browser (Zeiss GmbH, Jena, Germany), Corel Photopaint and CorelDRAW Graphics Suite (Corel Corporation, Ottawa, Ontario, Canada), and Adobe Photoshop (Adobe, San Jose, USA).

### Fluorimetric measurements of intracellular Ca^2+ ^levels

All physiological experiments were performed at the marine station Helgoland. The rhinophores were dissected as described above and cut longitudinally using a razor blaze. The half containing the ganglion was incubated for 60 min at 4°C with ASW containing 5 μM Fura II acetoxymethylester (AM). After removal of the incubation buffer the rhinophores were washed for 10 min. Changes in fluorescence were monitored with an imaging system (Visitron, Puchheim) and a CCD camera mounted on an inverted microscope (Zeiss Axiovert 100) equipped with a UV objective (Zeiss NeoFluar 20X). Different regions within the rhinophore ganglion were measured using the "region" function of the software (Metafluor, Meta Imaging Series, Universal Imaging Corporation). Changes in fluorescence were obtained by ratiometric measurements with excitation at 340 nm and 380 nm excitation. Values were presented as relative changes in ratios representing alterations in intracellular Ca^2+^-levels. Fluorescence images were acquired with an interval of 5 s and an exposure time of 50 ms per image.

For odour stimulation the recording chamber (volume 3 ml) was mounted on the microscope stage, and the bath flow was adjusted to 4 ml/min with a peristaltic pump. The chamber volume was exchanged in less than one minute. Amino acids, which induced the highest response in *Pleurobranchea californica *[32] were chosen: Alanine, Arginine, Glutamine, Glycine, Phenylalanine, Methionine and Valine. Amino acids (Sigma-Aldrich, Munich, Germany) were applied for one minute at various concentrations with the peristaltic pump system. In control experiments, the rhinophore ganglion was removed and treated similar as the rhinophore obtaining no response to amino acids. Each amino acid (1 M stock each) was dissolved in ASW and final concentrations ranged from 2 μM to 20 mM. Stimulus solutions were prepared immediately before use by dissolving the respective stock solution in ASW. After application the recording chamber was washed with ASW for at least 10 min to remove all amino acids possibly attached to the rhinophore. In most cases, 50 regions were measured simultaneously. As a control for the viability of the preparations the last stimulus at the end of an experiment was a high K^+ ^buffer stimulation (400 mM NaCl was replaced by 400 mM KCl), which always elicited a strong response. Calcium-imaging experiments were performed with 25 (K^+^)-responsive rhinophores.

## Authors' contributions

UB and WR had the general idea for this project and designed the neuroanatomical and neurophysiological experiments. AW performed the labelling with the fluorescent dyes and the fluorimetric measurements. MO did the histology. AW, WR and UB wrote the manuscript.
